# WBC-AMNet: Automatic classification of WBC images using deep feature fusion network based on focalized attention mechanism

**DOI:** 10.1371/journal.pone.0261848

**Published:** 2022-01-27

**Authors:** Ziyi Wang, Jiewen Xiao, Jingwen Li, Hongjun Li, Luman Wang

**Affiliations:** 1 College of Science, Beijing Forestry University, Beijing, China; 2 College of Environmental Science and Engineering, Beijing Forestry University, Beijing, China; 3 Department of Health Informatics and Management, Peking University Health Science Center, Beijing, China; COMSATS University Islamabad, Wah Campus, PAKISTAN

## Abstract

The recognition and classification of White Blood Cell (WBC) play a remarkable role in blood-related diseases (i.e., leukemia, infections) diagnosis. For the highly similar morphology of different WBC subtypes, it is too confused to classify the WBC effectively and accurately for visual observation of blood cell smears. This paper proposes a Deep Convolutional Neural Network (DCNN) with feature fusion strategies, named WBC-AMNet, for automatically classifying WBC subtypes based on focalized attention mechanism. To obtain more localized attention of CNN, the fusion features of the first and the last convolutional layer are extracted by focalized attention mechanism combining Squeeze-and-Excitation (SE) and Gather-Excite (GE) modules. The new method performs successfully in classifying monocytes, neutrophils, lymphocytes, and eosinophils on the complex background with an overall accuracy of 95.66%, better than that of general CNNs. The multi-classification accuracy of WBC-AMNet with the background segmentation is over 98% in all cases. In addition, Gradient-weighted Class Activation Mapping (Grad-CAM) is employed to visualize the attention heatmaps of different feature maps.

## Introduction

The analysis of White Blood Cell (WBC) images can assist clinical medicine experts in diagnosing many blood-related disorders such as leukopenia, Acute Leukemia (AL), agranulocytosis, etc. Importantly, AL is a malignant clonal disease of hematopoietic stem cells. Without special therapy, the average survival period is about three months, and even some patients died within a few days of diagnosis. AL is commonly classified into Acute Lymphoblastic Leukemia (ALL) and Acute Myelogenous Leukemia (AML) [1]. The survival rate of AML within five years is 40% [2], and in five cases in Europe, the annual survival rate of the disease is only 19% [3]. Therefore, the automated detection and classification of WBC sample images are of considerable reference value for leukemia diagnosis.

However, the presence of dyeing impurities and cytoplasm with low image contrast makes the microscopical differences between WBC more challenging to distinguish [4, 5].

In recent years, machine learning methods have been used for image classification of blood cells and have achieved excellent results in medicine. Standard high-performance classification methods and algorithms include Neural Network (NN), K-Nearest Neighbors (K-NN), Support Vector Machine (SVM), etc. The flexible neural tree algorithm of multi-classification NN cancer had an average accuracy rate of 98.6% on the mixed lineage leukemia [6]. The tumor diagnosis method based on the concept of biomarker association network could correctly classify all 72 samples for the WBC dataset [7]. Dichotomous classification of acute WBC samples achieved accuracy up to 86% [8]. Based on the multi-class SVM, an efficient hierarchical blood cell image recognition and classification method were proposed, with an average recall of 95.3% for six classifications of blood cells [9]. The advent of deep learning has led to experimentation with Convolutional Neural Network (CNN) in models for WBC classification [10].

At present, CNNs combining various methods have been successfully applied to the WBC classification [11, 12]. A recognition system, WBCsNet, based on deep convolution, was proposed to classify five categories on three different public WBC datasets with an accuracy of 96.1% [13]. A classification scheme involving CNN was proposed to classify 17092 images of normal peripheral blood cells with the best overall classification accuracy of 96.2% [14]. A CNN model with 32 feature maps achieved the accuracy of 88.25% and 81.74% in leukemia versus healthy and multi-classification of all subtypes, respectively [15]. Fifteen classifications were performed on 18365 peripheral blood smears using ResNeXt with an accuracy of more than 90% [16]. As powerful tools to assist physicians in diagnosing blood-related diseases, CNN algorithms still need further research on the generalizable properties and the explicit mechanisms of models detecting WBC images of blood smears.

Attention mechanism, as a standard feature extraction method, was widely used in deep learning and image classification in recent years due to its excellent performance and can be divided into Channel Attention Mechanism (CAM) and Spatial Attention Mechanism (SAM) [17]. We propose a method for automatically classifying WBC subtypes images in this paper. The new method, named WBC-AMNet, is based on attention mechanism and Deep CNN (DCNN), which well decreases the attention dispersing phenomenon due to the complex background of images.

The rest of this paper is presented below. Section 2 describes the material of WBC images in different contexts and elaborates the WBC-AMNet model in detail. The experimental results with different backgrounds and visual analysis are explored in section 3. A conclusion is drawn in the last section.

## Materials and methods

### WBC image datasets

In our experiment, the first WBC image dataset is from the Blood Cell Count Dataset (BCCD). BCCD contains the WBC images of eosinophils, monocytes, lymphocytes, and neutrophils cells. All images have complex background. Before our experiment, the enhanced 12515 images from BCCD have been divided into the training set, test set, and validation set. The details of the BCCD are described in Table 1.

**Table 1:** Descriptions of BCCD and WBCs dataset.

Dataset	Description	Category	Division	Subtypes	Number
BCCD	12515 images 320 × 240	4	training set (9957)	neutrophils	2499
				monocytes	2478
				lymphocytes	2483
				eosinophils	2497
			test set(2487)	neutrophils	624
				monocytes	620
				lymphocytes	620
				eosinophils	623
			validation set(71)	neutrophils	48
				monocytes	4
				lymphocytes	6
				eosinophils	13
WBCs Dataset	4358 raw images 112 × 112	4	-	neutrophils	2025
				monocytes	576
				lymphocytes	1586
				eosinophils	171

Another public WBC images dataset (WBCs dataset) from the Kaggle repository incorporates 4358 raw WBC images segmented in single cells, which eliminates the interference of complex background. The WBCs dataset has been labeled as seven different WBC subtypes, and four of them are used in our experiment (Table 1). Images from the WBCs dataset are in RGB color space, 112 × 112 pixels, and JPEG format. During the training of our model, the dataset is divided into 60% for training, 20% for validation, and 20% for testing.

**Fig 1:**
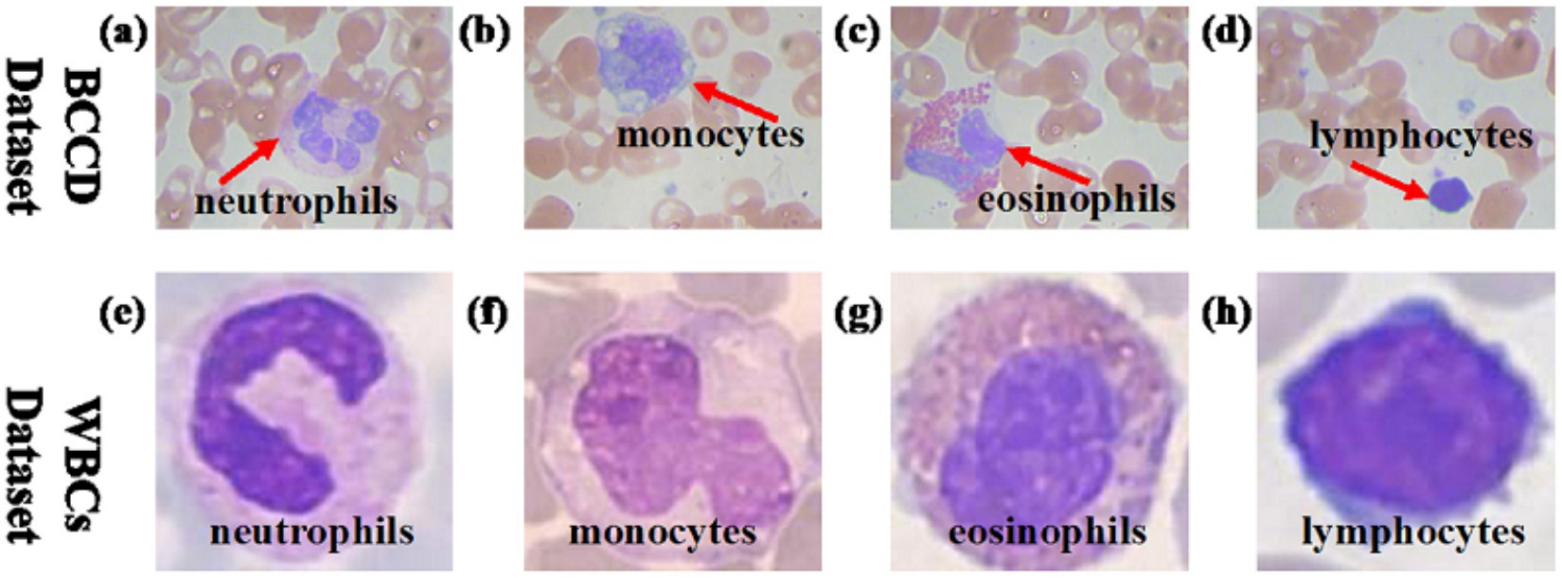
Sample images from BCCD (The first row) and the WBCs dataset (The second row). Among them, (a) and (e) are neutrophils, (b) and (f) are monocytes, (c) and (g) are eosinophils, and (d) and (h) are lymphocytes.

Several schematical images of two datasets with or without background segmentation are shown in Fig 1.

### Methods

The pipeline of our approach for classifying the WBC images are described following. In the first, the WBC images of the online source are taken from blood smears under microscopes and labeled by experts.

And then, the images are pre-processed: all images are resized to 224 × 224 pixels to fit the model; the random rotation (by an angle of -14 ∼ 15 degree), cropping, and flipping are used to eliminate the effects of irrelevant information and noise of images; and color distorting is conducted to make images clear. By color distorting, the brightness, contrast, saturation, and chromaticity of the image are adjusted by random factors taking values in (0, 1).

After that, the images are input to the proposed WBC-AMNet with a part of parameters pre-trained on the ImageNet dataset to train and fine-tune the model. The WBC-AMNet is implemented the proposed focalized attention mechanism, and Grad-CAM is conducted to visualize the attention.

**Fig 2:**
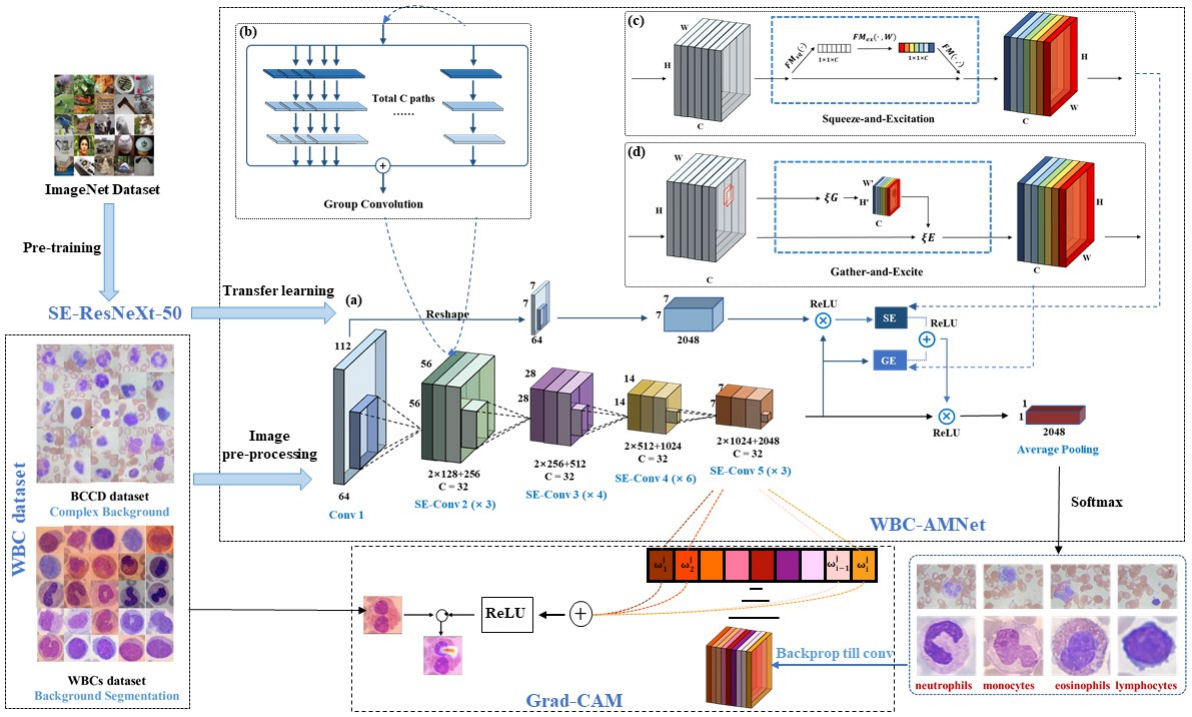
Flowchart of our method.

Finally, the classification results of WBC images are obtained and assist in diagnosing. Fig 2 depicts the flowchart of our method for the classification of different WBC subtypes.

At the training stage, the modes are performed on Baidu AI Studio platform with Tesla V100 GPU. The DCNNs model are implemented in the PaddlePaddle 1.7.2 deep learning framework of Python 3.7.

### WBC-AMNet model

Our WBC-AMNet model is based on DCNN architecture with focalized attention mechanism. DCNN architecture mainly refers the WBCsNet [13] and uses the group convolution strategy.

#### Group convolution strategy

The group convolution strategy was employed in ResNeXt, while adopting the idea of VGG stacking and split-transform-merge of Inception [18]. Group convolution improves the accuracy while reducing the number of hyperparameters, sketch map as Fig 2b. Group convolution can not only significantly reduce the amount of model calculations [19], but also improve the accuracy of WBC-AMNet.

The focalized attention mechanism is mainly realized by Squeeze-and-Excitation (SE) module and Gather-Excite (GE) module.

**Squeeze-and-Excitation (SE) module** is a computational module constructed based on the CAM [20]. The core idea of the SE module is to model the interdependencies between feature channels, namely, use the global average pooling to squeeze the WBC feature map, perform a nonlinear transformation by excitation, finally superimpose on the input features, and recalibrate the feature channels by adaptive learning. The structure of the SE module is shown in Fig 2c.

Let U = [u^1^, u^2^, …, u^C^] ∈ R^H×W×C^ be the input, and K = [k_1_, k_2_, …, k_c_] denote the kernel set of filters in learning, k_i_ denotes the parameter of the *i*–th filter $\left(k_{c}=[k_{c}^{1},k_{c}^{2},\ldots ,k_{c}^{C}]\right)$. After a series of transformations, we get F = [f_1_, f_2_, …, f_c_], then (Eq 1) [21]:

$$\begin{array}{c} f_{c}=k_{c}*U=\sum_{s=1}^{C}k_{c}^{s}u^{s} \end{array}$$ (1)

where * denotes convolution. Thus, we can obtain the result F after SE module (Eq 2):

$$\begin{array}{c} F\left(U\right)=\sum_{s=1}^{C}k_{c}^{s}\times u^{s}=\sum_{s=1}^{C}{\text{FM}}_{\text{scale}\;}(({\text{FM}}_{\text{ex}}({\text{FM}}_{\text{sq}}(u^{s}),W),u^{s}) \end{array}$$ (2)

Based on the SE module, the Gather-Excite (GE) further exploits the feature context in the CNN by introducing a pair of operators *ξ*G (step-wise deep convolution) and *ξ*E [22]. The core of the GE module is to use different filters layer by layer on the feature map, which makes WBC-AMNet aggregate the features extracted by WBC accordingly. Gather can effectively aggregate feature responses on a large spatial scale, and then excite is used to redistribute the aggregated information to local features. The structure of the GE module is displayed in Fig 2d. After processing by GE module, WBC-AMNet focuses on local features more precisely and improves the feature extraction ability greatly.

Let V be the output $\left(V\in R^{[\frac{H}{a}]\times [\frac{W}{\alpha }]\times C},v\in R^{\left[\frac{H}{a}]\times (\frac{W}{a}\right]}\right)$, then, the result G after GE module can be obtained as (Eq 3):

$$\begin{array}{c} G=\sum_{s=1}^{C}\xi E(u^{s},\xi G{\left(u\right)}_{v}^{s})=\sum_{s=1}^{C}\xi E(u^{s},\xi G(u^{s}⊙T_{\tau (v,\alpha )}^{s}))=\sum_{s=1}^{C}u^{s}⊙g(\xi G(u^{s}⊙T_{\tau (v,\alpha )}^{s})) \end{array}$$ (3)

where ⊙ is the hadamard product, *τ*(v, *α*) = {*α*v + *θ*: *θ* ∈ [−⌊*α* − 1], [2*α* − 1]]^2^/4}, *α*: the selected range ratio. T_{⋅}_ denotes the tensor and g is the well-defined mapping.

The fused features of the first and last convolutional layers in the model are input to the SE module, while the feature maps of the last convolutional layer are input to the GE module. Then, the features of the SE module and GE module are fused to obtain the attentional features, and the attentional features are finally fused with the original features of the last convolutional layer. The output of the model is depended on the type of WBC in the dataset. The method of fine-tuning is implemented to obtain the optimal parameters of WBC-AMNet by transfer learning and gradient learning rate strategy.

Fig 2a depicts the focalized attention mechanism with DCNN architecture for WBC image classification, where the ReLU activation function is ReLU(*x*) = max(0, *ω*^*T*^ *x* + *b*). ⊗ is an element-wise multiplication operator, namely, the input X and the input Y are multiplied element-by-element, and the output elements at each position are stored in the returned result, Out = *X* ⊗ *Y*. And ⊕ is an element-wise add operator, namely, the input X and the input Y are added element-by-element, and the output elements at each position are saved in the returned result, Out = X ⊕ Y.

The idea of focalized attention mechanism guides the WBC-AMNet construction base on the backbone of SE-ResNeXt implemented GE module and group convolution strategy (Table 2).

**Table 2:** Comparison of CNN structure between WBC-AMNet and other models.

stage	Output	ResNet-50 (32 × 4*d*)	SE-ResNeXt-50	WBC-AMNet	
conv1	112 × 112	7 × 7, 64, stride2	7 × 7, 64, stride2	7 × 7, 64, stride2	7 × 7, 2048, stride2
conv2	56 × 56	3 × 3, *max pool*, stride2	3 × 3, *max pool*, stride2	3 × 3, *max pool*, *stride*2	
		$[\begin{array}{c} 1\times 1,64\\ 3\times 3,64\\ 1\times 1,256 \end{array}]\times 3$	$[\begin{array}{c} 1\times 1,128\\ 3\times 3,128\\ 1\times 1,256\\ \text{fc},[16,256] \end{array}]\times 3$ C = 32	$[\begin{array}{c} 1\times 1,128\\ 3\times 3,128\\ 1\times 1,256\\ \text{fc},[16,256] \end{array}]\times 3$ C = 32	–
conv3	28 × 28	$[\begin{array}{c} 1\times 1,128\\ 3\times 3,128\\ 1\times 1,512 \end{array}]\times 4$	$[\begin{array}{c} 1\times 1,256\\ 3\times 3,256\\ 1\times 1,512\\ \text{fc},[32,512] \end{array}]\times 4$ C = 32	$[\begin{array}{c} 1\times 1,256\\ 3\times 3,256\\ 1\times 1,512\\ \text{fc},[32,512] \end{array}]\times 4$ C = 32	–
conv4	14 × 14	$[\begin{array}{c} 1\times 1,256\\ 3\times 3,256\\ 1\times 1,1024 \end{array}]\times 6$	$[\begin{array}{c} 1\times 1,512\\ 3\times 3,512\\ 1\times 1,1024\\ \text{fc},[64,1024] \end{array}]\times 6$ C = 32	$[\begin{array}{c} 1\times 1,512\\ 3\times 3,512\\ 1\times 1,1024\\ \text{fc},[64,1024] \end{array}]\times 6$ C = 32	–
conv5	7 × 7	$[\begin{array}{c} 1\times 1,512\\ 3\times 3,512\\ 1\times 1,2048 \end{array}]\times 3$	$[\begin{array}{c} 1\times 1,1024\\ 3\times 3,1024\\ 1\times 1,2048\\ \text{fc},[128,2048] \end{array}]\times 3$ C = 32	$[\begin{array}{c} 1\times 1,1024\\ 3\times 3,1024\\ 1\times 1,2048\\ \text{fc},[128,2048] \end{array}]\times 3$ C = 32	fc(SE(cov 1 + cov 5) + GE), [128, 2048]
				fc((SE(cov 1 + cov 5) + GE) + SE), [128, 2048]	
	1 × 1	global average pool 1000-d fc, softmax	global average pool 1000-d fc, softmax	global average pool 1000-d fc, softmax	

#### SE-ResNeXt

This module is used directly with residual networks of ResNeXt model to build SE-ResNeXt [20]. The innovation of the attention mechanism significantly improves the performance of the ResNeXt model with no additional calculative cost.

### Attention visualization

WBC-AMNet only outputs numerical results such as accuracy, but it is difficult to intuitively understand the essential features and locations that the model finally extracts. To explain the effect of focalized attention mechanism in more vivid detail, we visualize the feature extraction and attention heatmap of WBC-AMNet using the Grad-CAM method [23]. Assume that the penultimate layer produces m features maps F^m^(F^m^ ∈ R^H×W^ for any C) and $F_{ij}^{m}$ is the activation of *F*^*m*^ at location (i, j). Grad-CAM obtains the gradient information of the score *g*^*c*^ for class C and uses the average value of all gradients as the weight of the feature map. After weighting the extracted features, the ReLU operation finally highlights the crucial regions in the WBC images through the class-discriminative localization map Grad-CAM $L_{Grad-CAM}^{c}$ (Eq 4). Grad-CAM does not require retraining of the proposed model, and it visualizes the local position in the WBC image which allows WBC-AMNet make the final decision.

$$\begin{array}{c} L_{\text{Grad}-\text{CAM}}^{C}=\text{ReLU}(\sum_{m}(\frac{1}{M}\sum_{i}\sum_{j}\frac{\partial g^{C}}{\partial F_{\text{ij}}^{m}})F^{m}) \end{array}$$ (4)

### Evaluation

This paper analyzes the performance of classification using indexes including Accuracy (Eq 5), Specificity (Eq 6), Precision (Eq 7), and F1-score (Eq 8). They are calculated as follows:

$$\begin{array}{c} \;\text{Accuracy}\;=(\text{TP}+\text{TN})/(\text{TP}+\text{TN}+\text{FP}+\text{FN}) \end{array}$$ (5)

$$\begin{array}{c} \;\text{Specificity}\;=(\text{TN})/(\text{TN}+\text{FP}) \end{array}$$ (6)

$$\begin{array}{c} \;\text{Precision}\;=(\text{TP})/(\text{TP}+\text{FP}) \end{array}$$ (7)

$$\begin{array}{c} \;\text{F1-score}\;=(2\text{TP})/(2\text{TP}+\text{FN}+\text{FP}) \end{array}$$ (8)

Among them, True Positive (TP) indicates accurately identified positive data labels; False Positive (FP) indicates incorrectly identified positive data labels; True Negative (TN) indicates correctly identified negative data labels; False Negative (FN) indicates incorrectly identified negative data labels.

## Experiment

### Tri-classification results of BCCD

**Fig 3:**
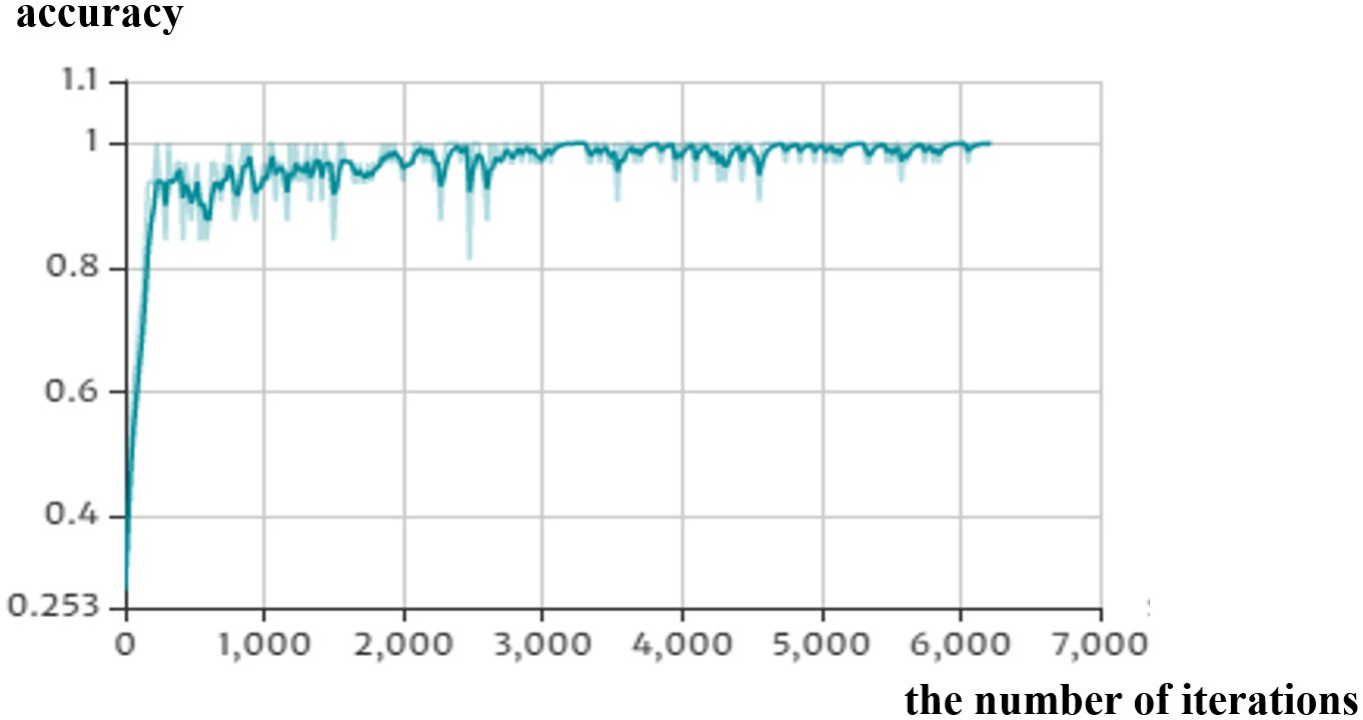
Classification accuracy versus the number of iterations in the training phase. (epoch = 20 and batch size = 32).

We perform a tri-classification analysis of WBC images from BCCD. Due to the presence of combined immune receptors based on T cell receptors in addition to T cells in neutrophils and monocytes, and they are derived from granulocyte monocyte progenitor cell [24, 35]. So we take monocytes and neutrophils cells as a set named MTD. The results of the search for epoch, batch size parameters, and the corresponding evaluation indexes are given in Table 3. Detailed data for WBC subtypes with different parameters are presented in S1 Table in S1 File. Fig 3 shows the change of objective function value in the training processing. As the number of iterations increases, the training accuracy improves rapidly in the initial stage, and then it converges to 1.00 gradually.

**Table 3:** Training results of tri-classification of BCCD images under different epoch and batch size.

Epoch	Batch size	WBC subtypes	Accuracy (%)	Specificity (%)	Precision (%)	F1-score (%)
15	32	3	90.71	90.71	90.74	90.70
20	16	3	94.93	94.94	95.03	94.93
**20**	**32**	**3**	**95.66**	**94.70**	**95.67**	**95.66**
25	32	3	95.13	95.13	95.14	95.11
30	32	3	93.81	93.81	93.97	93.69

Our model reaches optimal performance when epoch = 20 and batch size = 32, at which point the accuracy reached 95.66%. Under the optimal parameters, we analyze in detail the recognition and classification ability of WBC-AMNet for three WBC subtypes. In Table 4, the classification accuracy of lymphocytes is particularly outstanding, the precision of lymphocytes even reached 100%.

**Table 4:** Training results when epoch = 20 and batch size = 32.

WBC subtypes	Accuracy (%)	Specificity (%)	Precision (%)	F1-score (%)
lymphocyte	100.00	95.50	100.00	100.00
MTD	95.50	91.65	95.81	95.65
eosinophil	91.65	100.00	91.07	91.36
total	95.66	94.70	95.67	95.66

We use ROC curves and confusion matrices to visually show the classification performance of WBC-AMNet for each WBC subtype. The abscissa of the Receiver Operating Characteristic (ROC) curve is the FP Rate (FPR) and the ordinate is the TP Rate (TPR). The Area Under Curve (AUC) is defined as the area enclosed by the coordinate axis under the ROC curve. The larger the value of AUC, the better the performance of the model.

**Fig 4:**
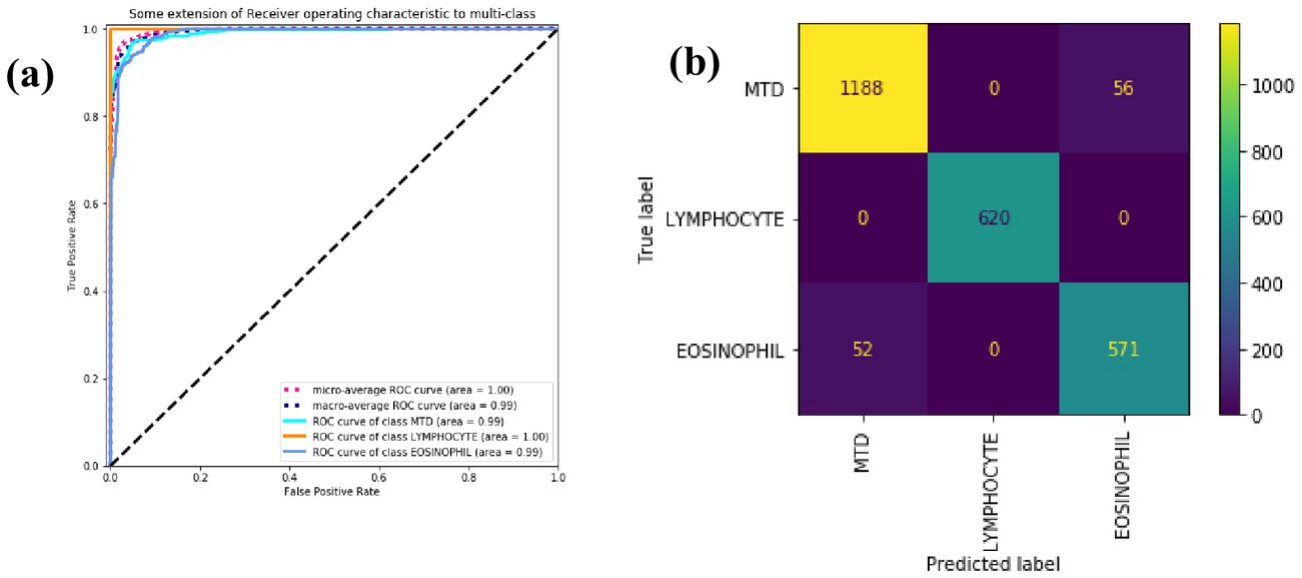
ROC curve and confusion matrix. (a) ROC curve of three subtypes of WBC. (b) Confusion matrix of three subtypes of WBC.

In Fig 4a, the solid lines of different colors represent the ROC curves of different WBC subtypes, and the blue dashed line represents the overall macroscopic average ROC curves. The AUC of the WBC-AMNet model is 0.99. All ROC curves demonstrate a high TPR and a low FPR. The ROC curves of each type are close to the upper left corner and far from the pure opportunity line. They indicate the solid tri-classification ability of the proposed model. The misclassification problem between MTD and eosinophils is reflected in the confusion matrix in Fig 4b. Both types of cells are easily confused with each other, whereas lymphocytes do not appear to be misclassified at all. The problem of misidentification between these two subtypes of WBC is also a common challenge for existing classifiers [11, 25].

We compare the tri-classification results obtained using our method with 10 general CNN models: VGG [26], ShuffleNetV2 [27], DPN [18], InceptionV4 [19], AlexNet [28], DistResNet [29], MobileNet-V1 [30], MobileNet-V2 [31], ResNet [29], SE-ResNeXt [32], as shown in Table 5. Detailed data for some of the models on the three WBC subtypes can be found in S2 Table in S1 File.

**Table 5:** Training results when epoch = 20 and batch size = 32.

ID	CNN model	Accuracy (%)	Specificity (%)	Precision (%)	F1-score (%)
1	VGG	50.02	33.33	16.67	22.23
2	ShuffleNetV2	79.41	83.33	81.81	80.99
3	DPN	87.45	90.73	87.82	88.15
4	InceptionV4	90.59	88.25	93.48	90.27
5	AlexNet	93.00	91.73	93.85	92.55
6	DistResNet	94.29	92.80	95.74	93.93
7	MobileNet-V1	94.17	93.60	94.63	94.07
8	MobileNet-V2	94.45	94.45	94.41	94.37
9	ResNet	93.12	93.13	93.32	93.17
10	SE-ResNeXt	93.93	93.93	94.07	93.97
11	WBC-AMNet	**95.66**	**94.70**	**95.67**	**95.66**

**Fig 5:**
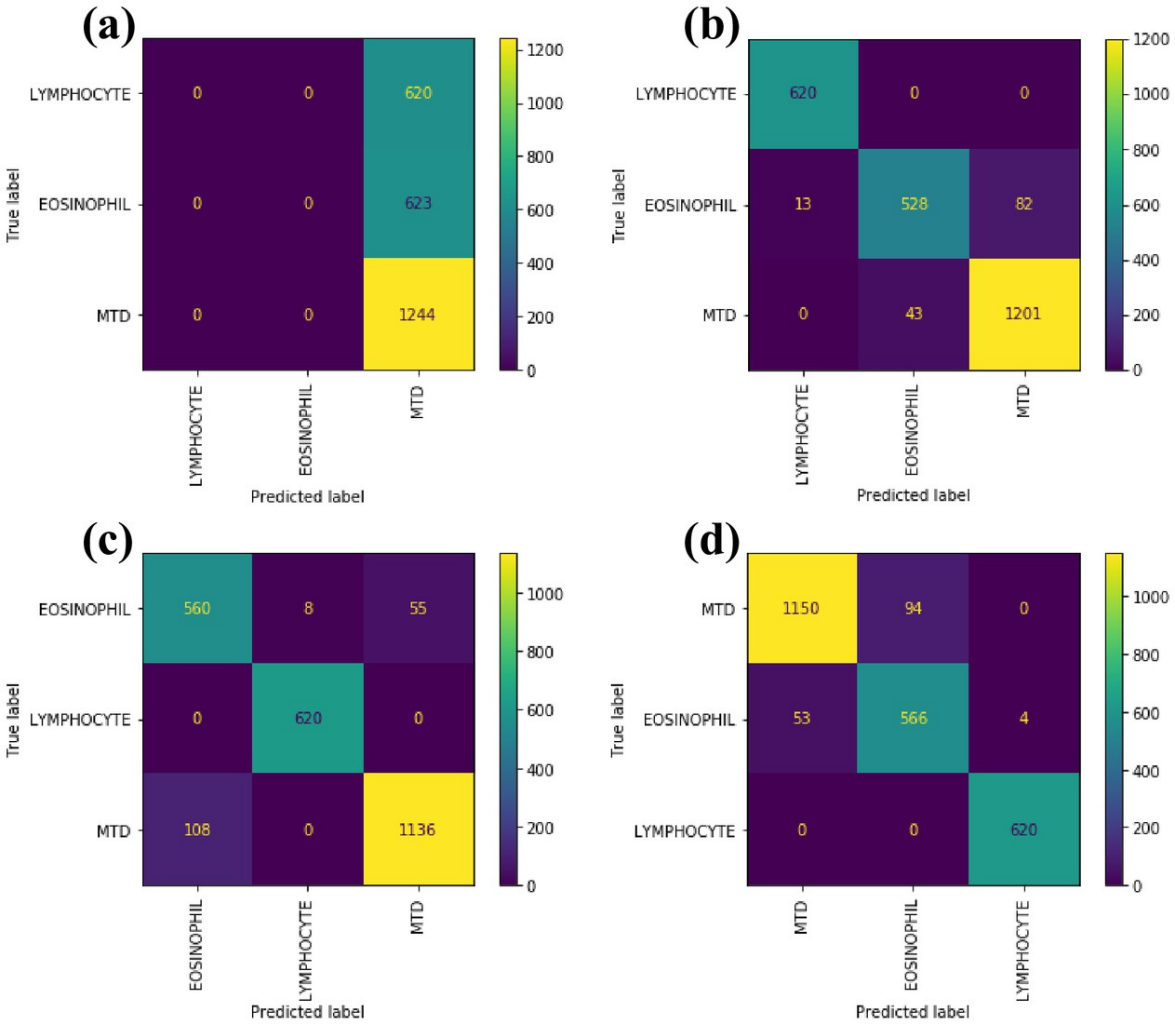
Confusion matrices of other CNN models. (a)VGG. (b)MobileNetV2. (c)ResNet. (d)SE-ResNeXt.

The accuracy of MobileNet-V1, MobileNet-V2, ResNet, DistResNet, and SE-ResNeXt are all over 93%, but the accuracy of WBC-AMNet is still significantly improved. The accuracy of WBC-AMNet is nearly two times higher than that of VGG, and other evaluation metrics also have significant differences. The simple structure of VGG makes it less practical for WBC classification in complex background. Compared with ResNet and SE-ResNeXt, the accuracy of the proposed model is improved by about 2%, which is a surprising result. The high accuracy and other statistics demonstrate that introducing the feature fusion strategy and the focalized attention mechanism in complex background can significantly and effectively improve the WBC-AMNet classification ability. To visually analyze the training results of other models, we compare the confusion matrices in Fig 5.

VGG (Fig 5a) is a conventional CNN model, which identifies all WBC subtypes as MTD. Compared with MobilNetV2 (Fig 5b), WBC-AMNet improves the problem of misclassifying eosinophils as MTD. It reduces the number of eosinophils misclassified by nearly one third. ResNet (Fig 5c) addresses the problem of misclassifying MTD as eosinophils to a certain extent. The introduction of the focalized attention mechanism allowed WBC-AMNet to target attention to valuable features. SE-ResNeXt (Fig 5d) improves the problem of misclassifying MTD as eosinophils and shows unexpected results in predicting MTD. The combination of focalized attention mechanism and feature fusion allows WBC-AMNet to obtain local attention moreover.

### Quad-classification results of BCCD

Monocytes and eosinophils are essential references for diagnosing diseases such as monocytic leukemia and an underlying allergic state, respectively [33, 34]. Accordingly, quad-classification is performed using WBC-AMNet for eosinophils, monocytes, lymphocytes, and neutrophils, with approximately 2480 training images and 620 test images for each subtype of WBC. Based on the results of the tri-classification parameter search, we refer to its optimal parameters (epoch = 20, batch size = 32), and the statistical results of different WBC subtypes are shown in Table 6. Due to a small proportion of monocytes and eosinophils are misclassified, resulting in their slightly lower accuracy. Detailed data for WBC subtypes with different parameters are presented in S3 Table in S1 File.

**Table 6:** Training results of different WBC subtypes.

WBC subtypes	Accuracy (%)	Specificity (%)	Precision (%)	F1-score (%)
eosinophils	82.50	82.50	91.46	86.75
neutrophils	93.43	93.43	73.70	82.40
monocytes	84.03	84.03	98.67	90.77
lymphocytes	96.94	96.94	99.17	98.04
total	89.22	89.22	90.72	89.48

Comparing the results in Table 4, we find that lymphocytes still maintain a high classification accuracy. However, after reclassifying the MTD, the accuracy of eosinophils decreased, and the accuracy of neutrophils and monocytes is also not high. We speculate that a misclassification problem occurred [35]. Although the accuracy of neutrophils is high, the predicted result is not as well as it should have, resulting in a low F1-score. Conversely, although the accuracy of monocytes is low, it is incredibly predictive. The above phenomenon indicates that WBC-AMNet has a good classification ability for neutrophils, but the precision is higher for monocytes.

**Fig 6:**
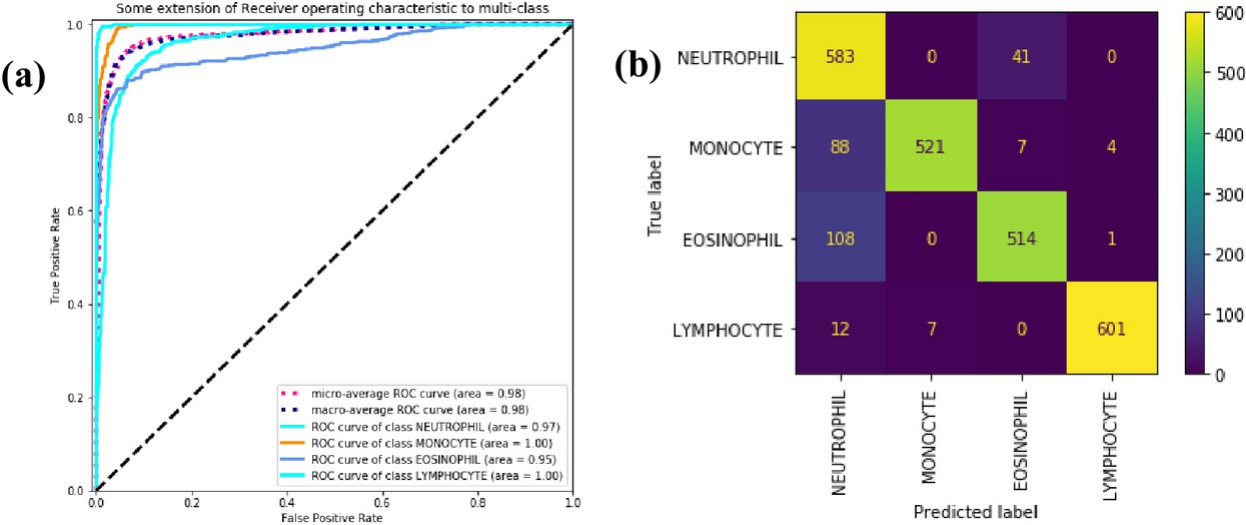
ROC curve and confusion matrix. (a) ROC curve of four subtypes of WBC. (b) Confusion matrix of four subtypes of WBC.

Fig 6a shows that the classification results are not satisfactory except for lymphocytes, reflected in the confusion matrix in Fig 6b. The quad-classification method identified monocytes and eosinophils as neutrophils several times, verifying our speculation. Other identification of WBCs as neutrophils is more numerous, but there are no cases of neutrophils identified as monocytes. Compared with monocytes, WBC-AMNet extracts the features of neutrophils more accurately.

Based on the 11 CNN models, the results and statistical data are shown in Table 7. Compared to Table 5, the classification ability of VGG is significantly improved on the quad-classification problems. The new model with the introduction of focalized attention mechanism has a significant improvement in accuracy compared to ResNet. The operation of feature fusion makes the classification accuracy of WBC-AMNet better than that of the best model SE-ResNeXt nowadays. Accuracy and other data intuitively reflect the important guiding significance of feature fusion for the model to extract features and process them.

**Table 7:** Statistical results of nine classic CNN models.

ID	CNN model	Accuracy (%)	Specificity (%)	Precision (%)	F1-score (%)
1	AlexNet	82.31	82.32	86.12	82.70
2	ShuffleNetV2	83.43	83.43	85.80	83.53
3	DPN	84.84	84.84	87.50	85.28
4	VGG	86.81	86.81	88.49	87.03
5	DistResNet	87.74	87.73	89.95	88.02
6	InceptionV4	87.94	87.94	90.41	88.23
7	MobileNet-V1	86.13	86.13	88.96	86.48
8	MobileNet-V2	88.82	88.82	90.85	89.02
9	ResNet	86.65	86.65	88.08	86.81
10	SE-ResNeXt	87.78	87.78	89.43	87.91
11	WBC-AMNet	**89.22**	**89.22**	**90.72**	**89.47**

### Classification results of WBCs dataset

We use our method to classify the WBC images from WBCs dataset. These WBC images are all without complex background. In this section, we compare our method with 3 representative methods: From Tables 5 and 7, it can be found that MobileNetV2 has a higher accuracy rate. Comparing ResNet and SE-ResNeXt with WBC-AMNet, respectively, we will get the effect of introducing attention mechanism and GE module. On the premise of the same parameters (epoch = 20, batch size = 32), we choose MobileNetV2, ResNet and SE-ResNeXt to train and compare them with WBC-AMNet.

#### Tri-classification of WBCs dataset

**Fig 7:**
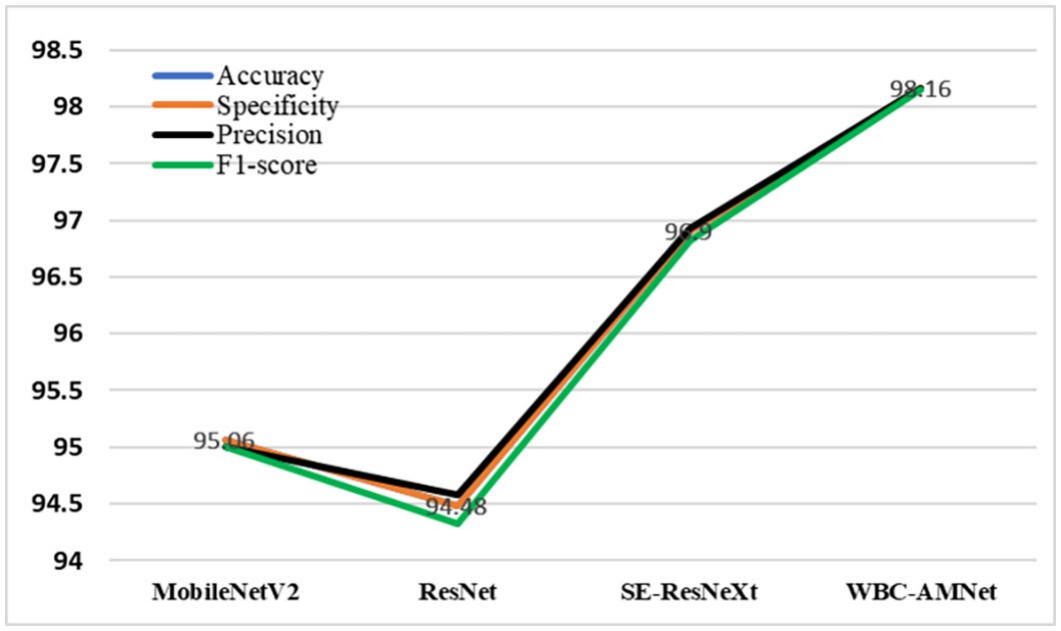
Tri-classification line chart of WBCs dataset.

First, the tri-classification results of different WBC subtypes are analyzed in Table 8. Slightly different from the BCCD, WBC-AMNet has a higher classification accuracy for neutrophils in the WBCs dataset, regardless of the model. Except for the intermediate cell, the classification accuracy of WBC-AMNet is above 99%, which is a satisfactory result. However, the accuracy of the intermediate cell is slightly lower, and we will reclassify it in the next section to further explore the reason. As shown in Fig 7, our model has the best performance among 4 methods in the view of 4 indexes: Accuracy, Specificity, Precision and F1-score.

**Table 8:** Tri-classification results of images from WBCs dataset.

ID	CNN model	WBC subtypes	Accuracy(%)	Specificity(%)	Precision(%)	F1-score (%)
1	MobileNetV2	intermediate cell	84.46	84.46	89.29	86.81
		lymphocyte	94.75	94.65	96.78	95.71
		neutrophil	99.26	99.26	95.70	97.45
		total	95.06	95.06	95.00	95.00
2	ResNet	intermediate cell	77.70	77.70	95.04	85.50
		lymphocyte	95.91	95.91	96.83	96.37
		neutrophil	99.51	99.50	92.63	95.94
		total	94.48	94.48	94.58	94.32
3	SE-ResNeXt	intermediate cell	85.14	85.14	97.67	90.97
		lymphocyte	98.74	98.74	95.15	96.91
		neutrophil	99.75	99.75	98.05	98.90
		total	96.90	96.90	96.93	96.82
4	WBC-AMNet	intermediate cell	93.24	93.24	97.18	95.17
		lymphocyte	99.37	99.37	97.23	98.29
		neutrophil	99.01	99.01	99.26	99.13
		total	**98.16**	**98.16**	**98.16**	**98.15**

The accuracy of WBC-AMNet combined with the focalized attention mechanism is nearly 4% higher than that of ResNet. WBC-AMNet also achieves more than 1% higher accuracy than SE-ResNeXt, not only due to the introduction of GE module but also thanks to the operation of feature fusion. Regardless of the model, the classification accuracy of intermediate cells is lower than that of other cells. But for lymphocytes, the accuracy of the proposed model has increased, which is the main reason for the increase in total accuracy.

**Fig 8:**
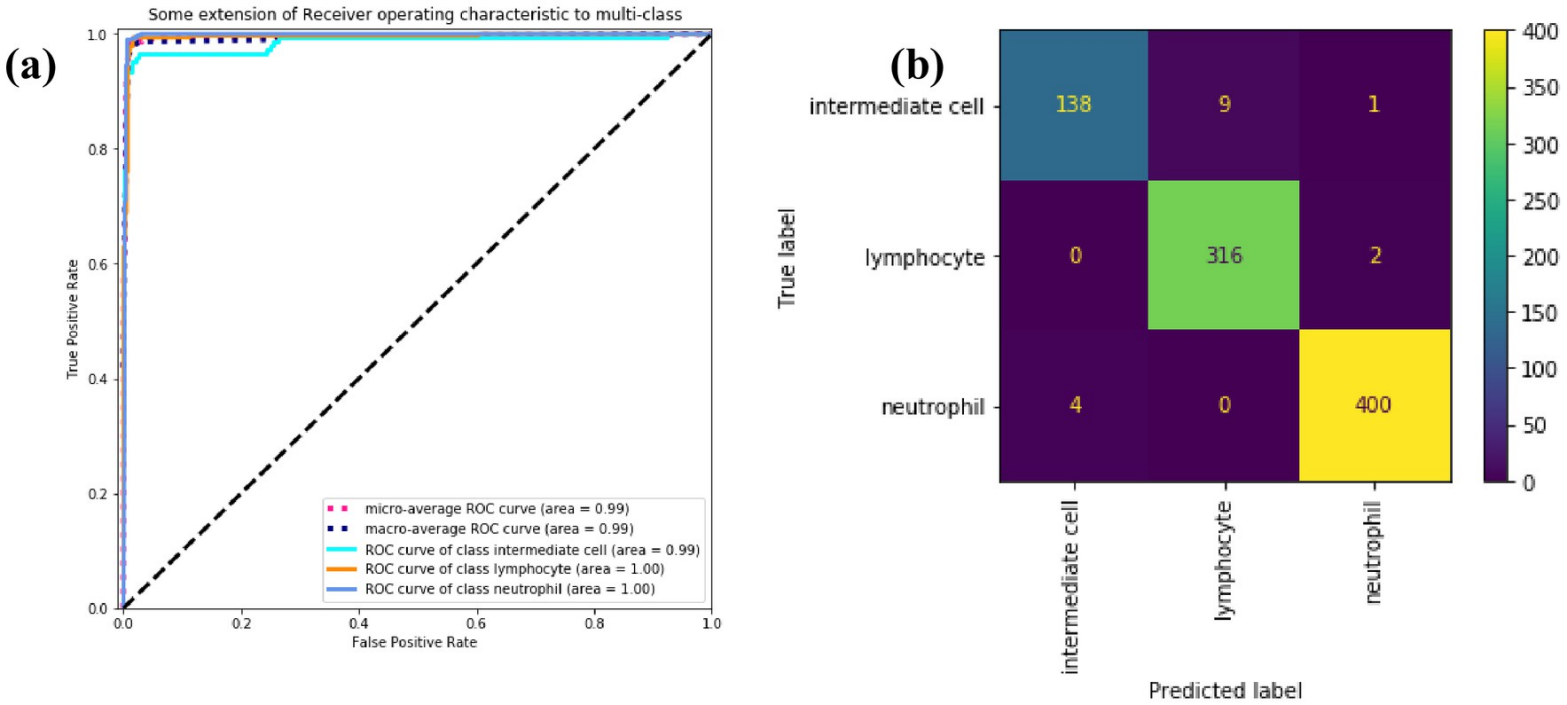
ROC curve and confusion matrix. (a) ROC curve of three subtypes of WBC. (b) confusion matrix of three subtypes of WBC.

The AUCs of lymphocytes and neutrophils in Fig 8 are both 1.00, and the AUC of intermediate cells and the whole is 0.99. From Fig 8b, we can observe that a tiny number of intermediate cells (i.e. MTD) are still misclassified as lymphocytes, which is different from the conclusion of the BCCD. We suspect believed to be caused by different problems in different contexts misclassification. The confusion matrices of the four models depict in S5 Fig in S1 File.

**Fig 9:**
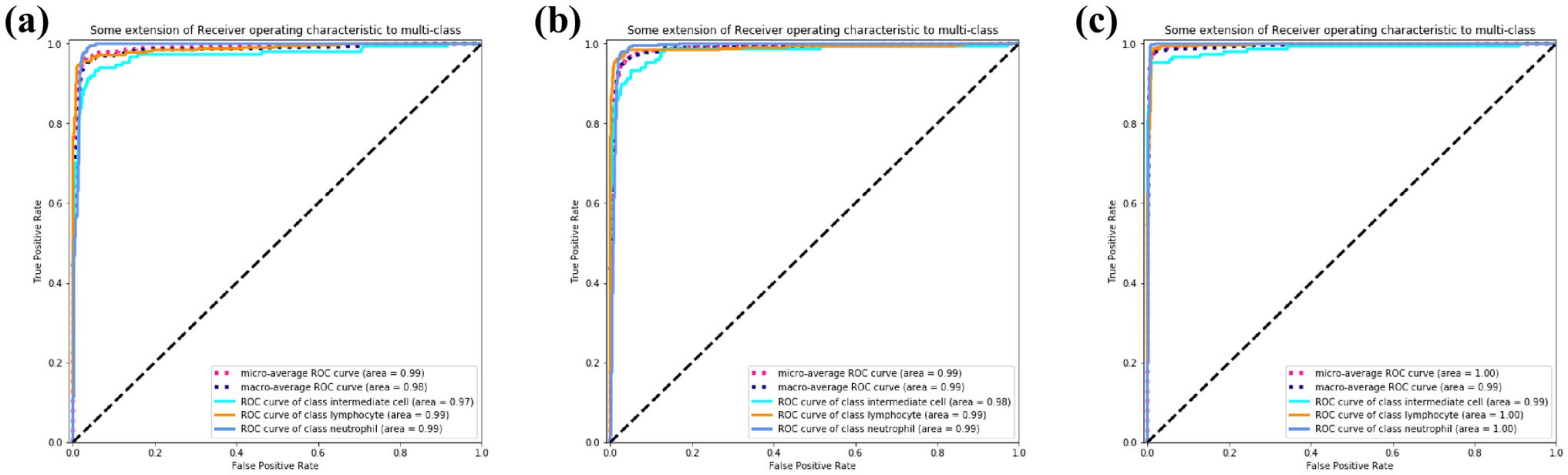
ROC curve. (a) MobileNetV2. (b) ResNet. (c) SE-ResNeXt.

In Fig 9, the solid lines of different colors represent the ROC curves of different WBC subtypes, and the blue dashed line represents the overall macroscopic average ROC curves. The AUC of MobileNetV2 (Fig 9a) is 0.98, the AUC of ResNet and SE-ResNeXt is 0.99. WBC-AMNet improves the classification of all three WBC subtypes. From Fig 9a and 9b, the light blue curve is slightly lower, which intuitively shows that the classification capabilities of MobileNetV2 and ResNet for intermediate cells are little poor. Comparing Figs 8a to 9, SE-ResNeXt has improved over the previous two models, and WBC-AMNet is more stable for the classification of intermediate cells.

#### Quad-classification of WBCs dataset

The classification results and statistics of our method and three compared methods for the four WBC subtypes are listed in Table 9. The classification rate of WBC-AMNet in eosinophils becomes almost twice as high as that of MobileNetV2 and ResNet. Since both triple and quadruple classifications are improved, we conclude that WBC-AMNet gets the best performance among four methods.

**Table 9:** Quad-classification results of images from WBCs dataset.

ID	CNN model	WBC subtypes	Accuracy(%)	Specificity(%)	Precision(%)	F1-score (%)
1	MobileNetV2	eosinophils	50.00	50.00	77.27	60.71
		neutrophils	98.02	98.02	95.66	96.83
		monocytes	69.82	69.83	84.38	76.42
		lymphocytes	97.79	97.78	91.42	94.50
		total	92.31	92.30	91.90	91.86
2	ResNet	eosinophils	61.76	61.76	91.30	73.68
		neutrophils	98.77	98.77	95.47	97.09
		monocytes	84.48	84.48	87.50	85.96
		lymphocytes	96.20	96.20	95.90	96.05
		total	94.49	94.49	94.40	94.32
3	SE-ResNeXt	eosinophils	97.06	97.06	97.06	97.06
		neutrophils	99.75	99.75	98.78	99.26
		monocytes	85.34	85.34	92.52	88.79
		lymphocytes	97.79	97.78	96.26	97.02
		total	97.02	97.01	96.96	96.97
4	WBC-AMNet	eosinophils	97.06	97.06	97.06	97.06
		neutrophils	99.75	99.75	99.02	99.38
		monocytes	95.69	95.69	97.37	96.52
		lymphocytes	97.78	97.78	98.10	97.94
		total	**98.39**	**98.39**	**98.39**	**98.39**

**Fig 10:**
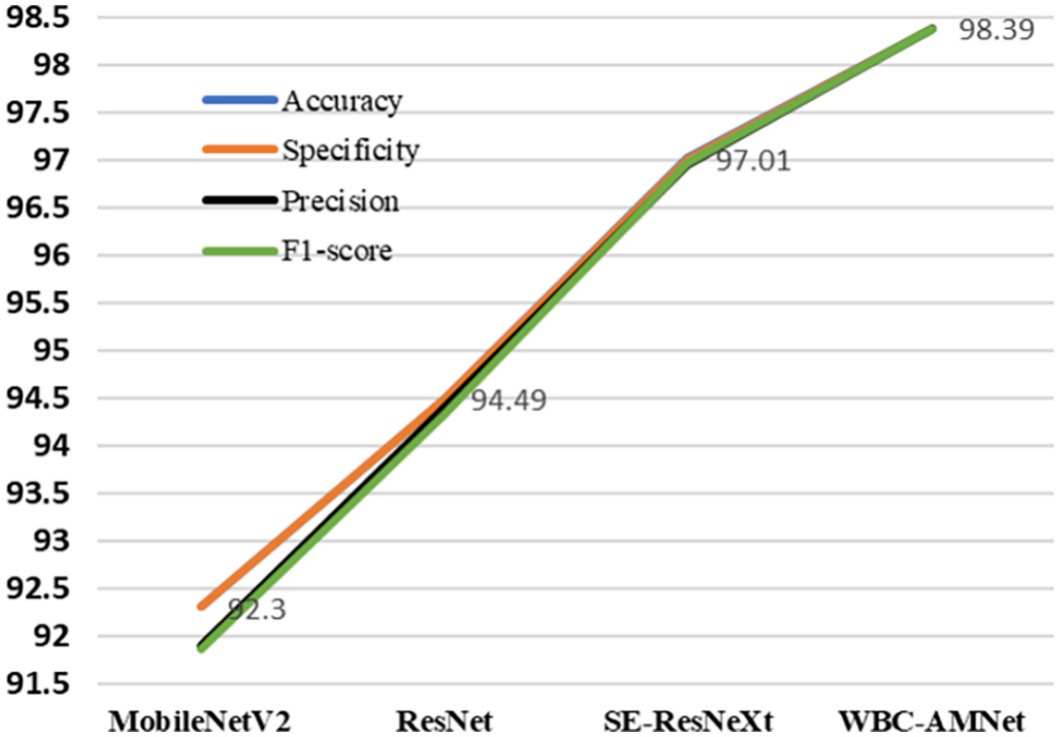
Quad-classification line chart of WBCs dataset.

MobileNetV2 has serious misclassification problems when recognizing eosinophils and monocytes, resulting in a low accuracy of these two subtypes of WBC. Except for eosinophil, ResNet has a higher accuracy for other WBC subtypes. However, since the number of images of eosinophils in the WBCs dataset is small, it has little effect on the overall accuracy. With the SE module, the accuracy of SE-ResNeXt has been significantly improved. Especially for eosinophils, SE-ResNeXt is about 35% higher than ResNet. Such a large increase in accuracy verifies the importance and effectiveness of using the attention mechanism strategy. The classification accuracy of monocytes is still not satisfactory. By integrating the GE module, the recognition accuracy of WBC-AMNet in monocyte is improved by nearly 10% compared with SE-ResNeXt. Moreover, WBC-AMNet has an accuracy rate of over 95% for each WBC subtype. So far, we can come to the conclusion: WBC-AMNet has achieved effective WBC classification on WBCs dataset. Fig 10 shows that for quad-classification, our model, comparing to other 3 methods, still gets the higher scores of Accuracy, Specificity, Precision and F1-score.

**Fig 11:**
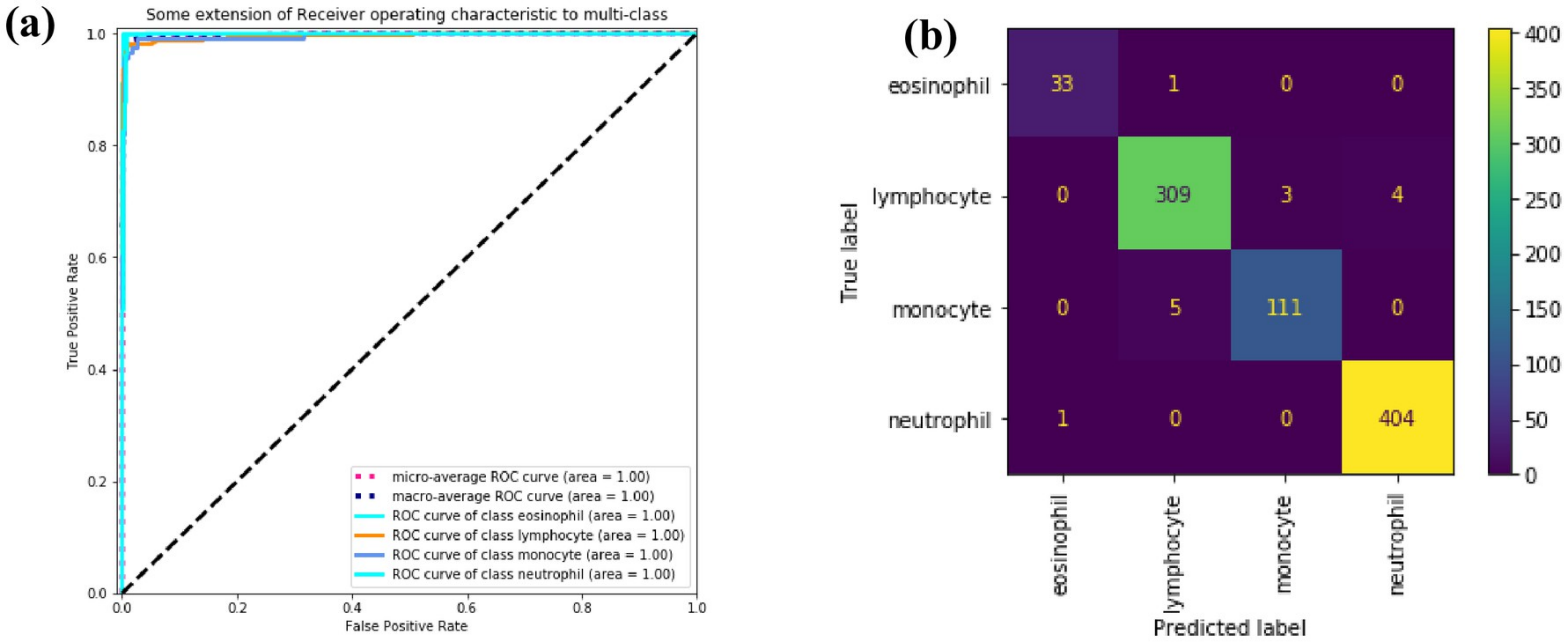
ROC curve and confusion matrix. (a) ROC curve of four subtypes of WBC. (b) Confusion matrix of four subtypes of WBC.

In Fig 11a, the AUC value of 1.00 is reached for all WBC subtypes. There is almost no misclassification problem in Fig 11b. The confusion matrices of the four models are demonstrated in S6 Fig in S1 File.

**Fig 12:**
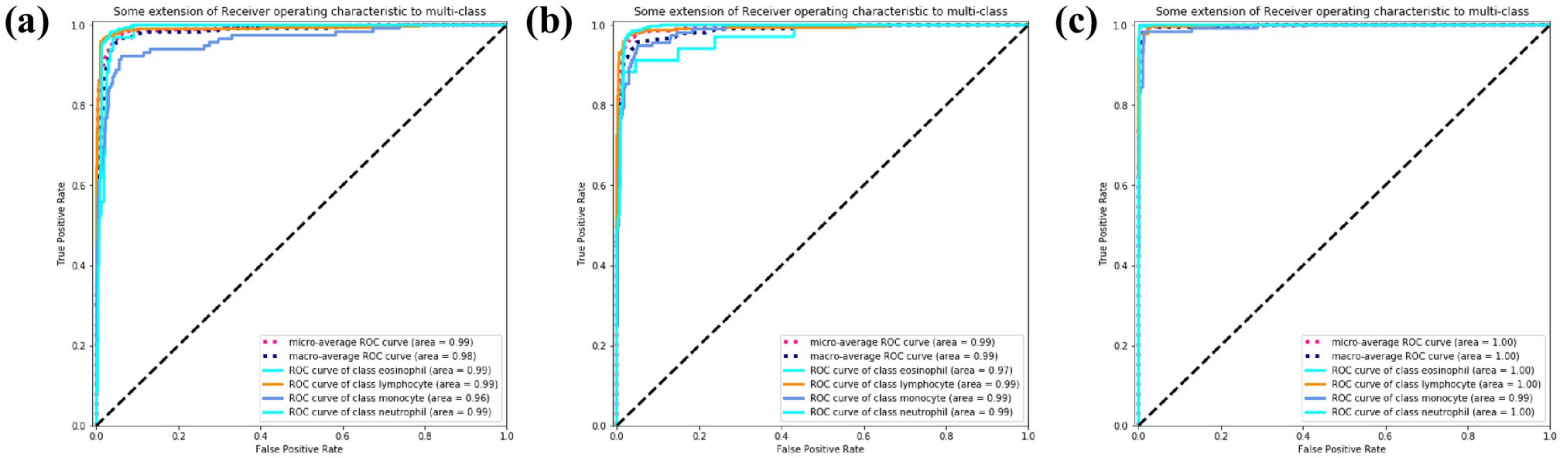
ROC curve. (a) MobileNetV2. (b) ResNet. (c) SE-ResNeXt.

In Fig 12, MobileNetV2 has an AUC of 0.98, ResNet has an AUC of 0.99, SE-ResNeXt and WBC-AMNet has an AUC of 1.00. The closer the AUC is to 1.00, the better the performance of the model. It can be seen from AUC that the performance of the model is improved after introducing the attention mechanism. Comparing Fig 12 with Fig 11a, the AUCs of MobileNetV2 and ResNet are lower on eosinophils and monocytes, the ROC of SE-ResNeXt on monocytes is slightly lower, and WBC-AMNet has reached 1.00 on all WBC subtypes, which means that WBC-AMNet is significantly improved compared to other CNN models.

### Visualization analysis

**Fig 13:**
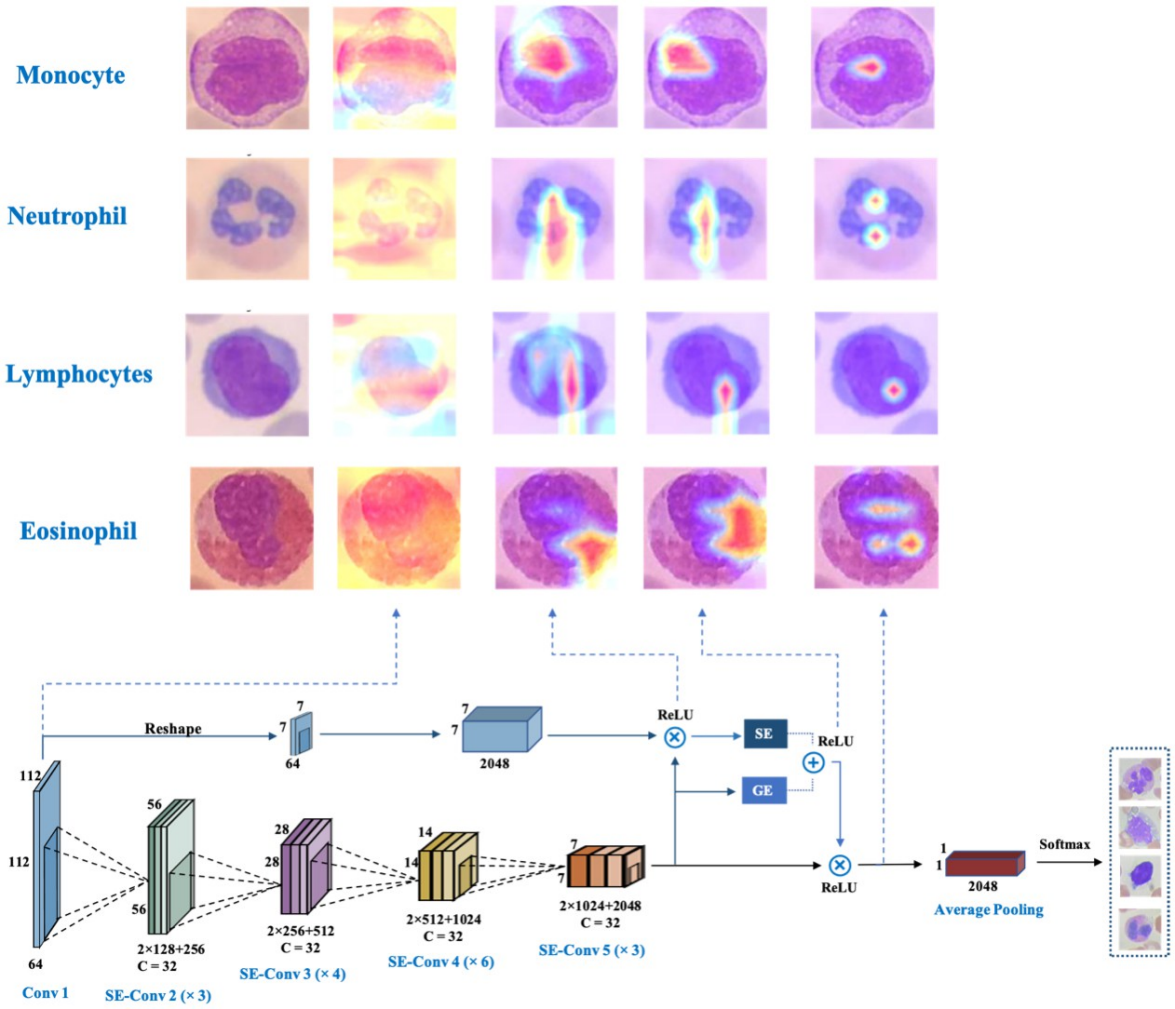
WBC-AMNet visualization analysis of attention to different feature maps.

The attention of different feature maps of WBC-AMNet is visualized in the background of single-cell segmentation. Firstly, the heatmap is obtained by a regular convolution operation. The first heatmap has highlighted regions spread over almost the whole WBC image and is very distracting. In order to focus the attention, the strategies of focalized attention mechanism and feature fusion are further introduced. The first and last convolutional layers in the model are feature fused and fed into the SE module. At this point, the area of the highlighted region in the heatmap is significantly reduced, and the red part starts to accumulate in the cell nucleus. Then, the feature map of the last convolutional layer is input to the GE module and fused with the features of the SE and GE modules to obtain the attentional features. The attentional aggregation is slightly reduced, and almost all of them are on the WBC nuclei. Finally, the attention features are fused with the original features of the last convolutional layer. The final heat map obtained reflects the superiority of the WBC-AMNet model. By implementing focalized attention mechanism and deep feature fusion, attention is highly focused on vital and partial locations of the WBC nuclei. Our proposed method extracts the effective critical information in the WBC cell nuclei and avoids the influence of too much redundant and invalid information on the results (Fig 13).

## Conclusion

In this paper, we propose a new DCNN, WBC-AMNet, for automatic classification of WBC images based on focalized attention mechanism and deep feature fusion strategy. The attention of different feature maps of WBC-AMNet is visualized using the Grad-CAM method, which extracts the critical practical information from the WBC cell nuclei and avoids the influence of too much redundant and invalid information on the results. Experimental results show that WBC-AMNet gets the better performance than that of several existing models. Although the classification effect of our model is satisfactory, the mathematical mechanism of network architecture is still unclear. In the future, we intend to study the deep learning network from the perspective of mathematics and test more medical image data using our model.

## Supporting information

S1 File Supplementary material. (PDF)
