# Supplementary material for: WBC-AMNet: Automatic classification of WBC images using deep feature fusion network based on focalized attention mechanism
Source: PLoS One. 2022 Jan 27;17(1):e0261848. doi: 10.1371/journal.pone.0261848 (PMC8794158; doi:10.1371/journal.pone.0261848)
Supplement: S1 File — (PDF) [file pone.0261848.s001.pdf]

Supplementary material

Image pre-processing

Since the quality of the WBC images captured by the camera is not up to the desired standard, image pre-processing is performed to improve the clarity and visibility of the input WBC images. The WBC images are pre-processed with forced image scaling size, random cropping, and image enhancement. The image size was similarly scaled to 224×224 pixels to fit the model. The feature map of the last convolutional layer in WBC-AMNet is set to 7×7. If the size is too small, it will lead to profound information loss, and if the size is too large, the amount of calculation will increase rapidly. Furthermore, the image resolution usually varies in multiples of 2. Then we find $7 \times 2^5 = 224$, which is the closest value to the 300-explanation required by the proposed model. In order to prevent irrelevant information in the image from affecting the classification results, we use random cropping to eliminate image noise. Random cropping of images not only increases the amount of data but also weakens the data noise. A uniformly distributed random number generator determines the cropping position. The random cropping operation increases the stability of the model by attenuating the weight of the noise factor. Image enhancement performs random rotation, brightness adjustment, contrast adjustment, saturation adjustment, and chromaticity adjustment on the WBC images to prevent overfitting and enhance discrimination. The image is randomly rotated by an angle of [-14,15) using a random number generator. The brightness, contrast, saturation, and chromaticity of the image are adjusted by random factors taking values in (0, 1). The images of WBC after different pre-processing are depicted in S1 Fig.

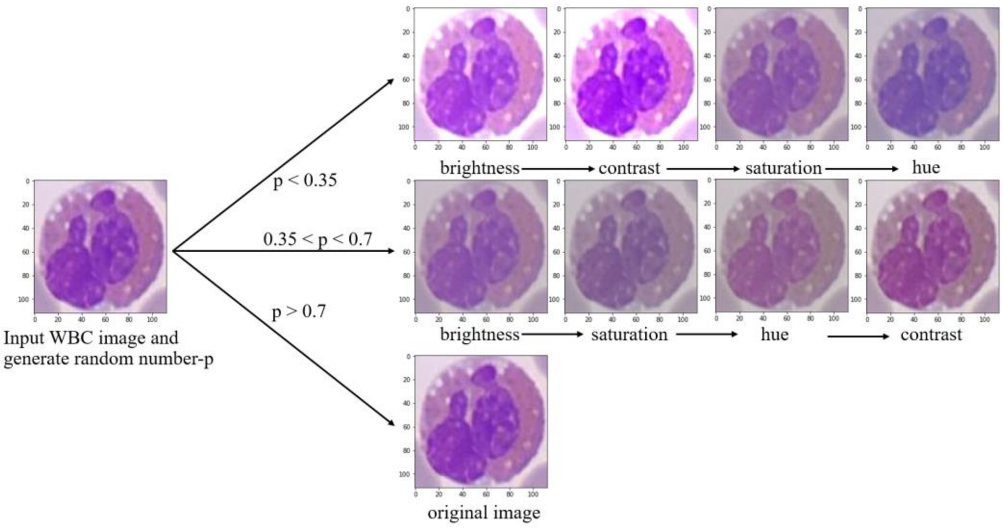

S1 Fig. Image pre-processing.

Model Comparison

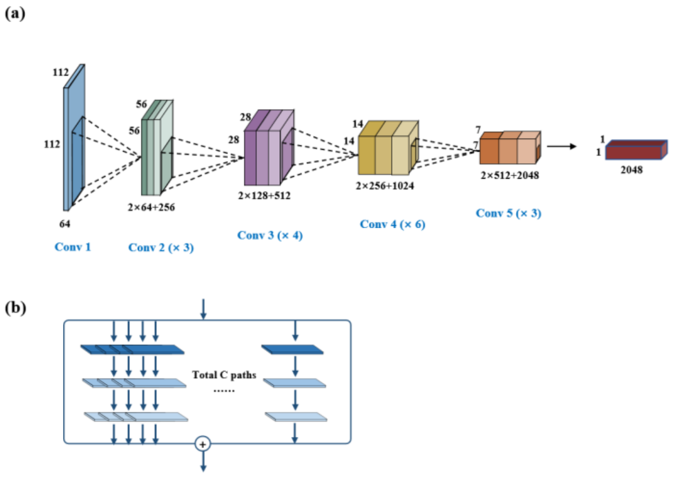

S2 Fig. The structure of ResNet model. (a) ResNet. (b) Group convolution.

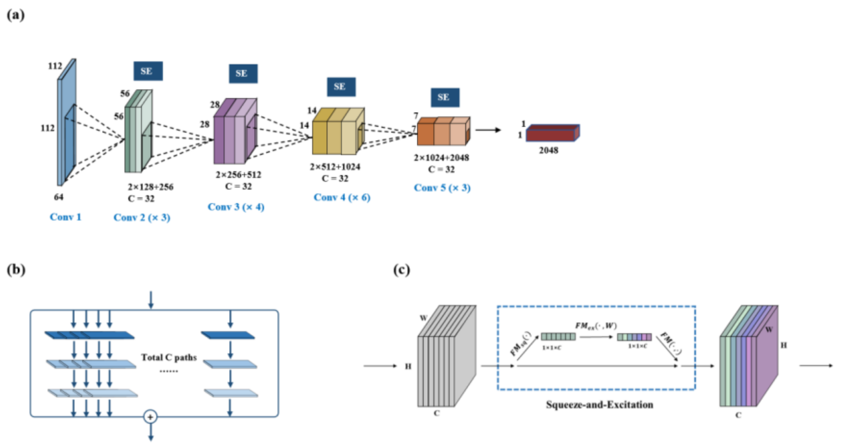

S3 Fig. The structure of the proposed SE-ResNeXt model. (a) SE-ResNeXt. (b) Group convolution. (c) SE module.

WBC classification tasks of BCCD

Tri-classification task

The classification results of WBC-AMNet for three WBC subtypes with different epoch and batch size are shown in detail in S1 Table. When the batch size is kept constant, the accuracy reaches the maximum at epoch = 20 with the epoch increase. At this time, the accuracy of all three WBCs is improved, but lymphocytes are the most obvious. When keeping the epoch constant, the accuracy reached the maximum at batch size = 32 with the increase of batch size. At this time, the accuracy of intermediate cells decreased slightly, but the other two WBCs increased more significantly. Therefore, we consider epoch = 20 and batch size = 32 as the optimal parameters.

S1 Table. WBC-AMNet tri-classification training results under different epoch and batch size.

Epoch	Batch size	WBC subtypes	Accuracy (%)	Specificity (%)	Precision (%)	F1-score (%)
15	32	lymphocytes	90.32	90.32	94.59	92.41
		eosinophils	86.04	86.04	87.44	86.73
		MTD	93.25	93.25	90.48	91.84
		total	90.71	90.71	90.74	90.70
**20**	**32**	lymphocytes	100.00	95.50	100.00	100.00
		eosinophils	91.65	100.00	91.07	91.36
		MTD	95.50	91.65	95.81	95.65
		total	**95.66**	**94.70**	**95.67**	**95.66**
25	32	lymphocytes	98.70	98.71	100.00	99.35
		eosinophils	88.60	88.60	92.93	90.71
		MTD	96.62	96.62	93.83	95.21
		total	95.13	95.13	95.14	95.11
20	16	lymphocytes	95.32	95.32	99.83	97.52
		eosinophils	89.73	89.73	94.43	92.02
		MTD	97.35	97.35	92.94	95.09
		total	94.93	94.94	95.03	94.93

S4 Fig showed the training process of WBC-AMNet under the optimal parameters. S4.a Fig portrayed the results of the training loss, which displayed the opposite trend of the training accuracy and eventually converged to 0.00. The training loss also proved that the error between the predicted and actual training results in the WBC-AMNet model was tiny. S4.b Fig showed the testing accuracy, which constantly fluctuated in the range of 0.90 and above, showing that the proposed model had a high recognition accuracy. In the training process, the training accuracy of the new model exceeded 95% at 200 steps, and the training loss also reached a low level. With the increase of steps, WBC-AMNet showed a more robust and powerful image classification ability.

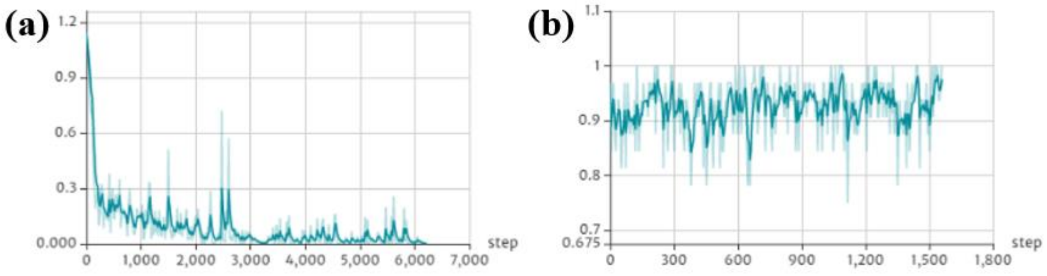

S4 Fig. Model training process (*epoch* = 20 and *batch size* = 32). (a) training loss. (b) testing accuracy.

The details of the tri-classification of WBC by three different CNN models are shown in detail in S2 Table. All three CNN models achieve a satisfactory classification rate for lymphocytes, and WBC-AMNet offers a clear advantage on intermediate cells. We can assume that the introduction of deep feature fusion and focalized attention mechanism makes WBC-AMNet stands out. In the complex context, the focalized attention mechanism makes the proposed model focus more on the features of the cell nucleus, which improves the classification accuracy.

S2 Table. Statistics of different CNN models for three WBC subtypes

ID	CNN model	WBC subtypes	Accuracy (%)	Specificity (%)	Precision (%)	F1-score (%)
1	ResNet	lymphocytes	100.00	100.00	98.73	99.36
		eosinophils	89.89	89.89	83.83	86.75
		MTD	91.32	91.32	95.38	93.31
		total	93.12	93.13	93.32	93.17
2	SE-ResNeXt	lymphocytes	100.00	100.00	99.36	99.68
		eosinophils	90.85	90.85	85.76	88.23
		MTD	92.44	92.44	95.59	93.99
		total	93.93	93.93	94.07	93.97
3	WBC-AMNet	lymphocytes	100.00	95.50	100.00	100.00
		eosinophils	91.65	100.00	91.07	91.36
		MTD	95.50	91.65	95.81	95.65
		total	**95.66**	**94.70**	**95.67**	**95.66**

Quad-classification task

The intermediate cells are further subdivided in the complex context, and the quad-classification is slightly less effective than the tri-classification. We can find that the classification accuracy of monocytes is significantly lower than the other three cells. However, the accuracy of lymphocytes is still high, but we suppose that the misclassification problem occurred. WBC-AMNet improved almost 10% over ResNet for neutrophils, which directly illustrates the rationale and importance of introducing attention mechanism. (S3 Table).

S3 Table. Quad-classification results of three models

ID	CNN model	WBC subtypes	Accuracy (%)	Specificity (%)	Precision (%)	F1-score (%)
1	ResNet	eosinophils	89.57	89.57	81.7	85.45
		neutrophils	**82.37**	82.37	71.89	76.77
		monocytes	75.00	75.00	99.36	85.48
		lymphocytes	99.68	99.68	99.52	99.60
		total	86.65	86.65	88.08	86.81
2	SE-ResNeXt	eosinophils	89.09	89.09	85.38	87.20
		neutrophils	88.78	88.78	72.70	79.94
		monocytes	73.87	73.87	99.78	84.89
		lymphocytes	99.35	99.35	100.00	99.68
		total	87.78	87.78	89.43	87.91
3	WBC-AMNet	eosinophils	82.50	82.50	91.46	86.75
		neutrophils	**93.43**	93.43	73.70	82.40
		monocytes	84.03	84.03	98.67	90.77
		lymphocytes	96.94	96.94	99.17	98.04
		total	89.22	89.22	90.72	89.48

WBC classification tasks of WBCs dataset

Tri-classification task

The values of accuracy and other descriptive statistics are improved in a single-cell scene, excluding the interference of other cells and the external environment. S5 Fig depicts the confusion matrices of 4 types of CNN models. The misclassification problem still exists, but WBC-AMNet has improvement in classification accuracy due to the introduction of focalized attention mechanism and deep feature fusion strategies.

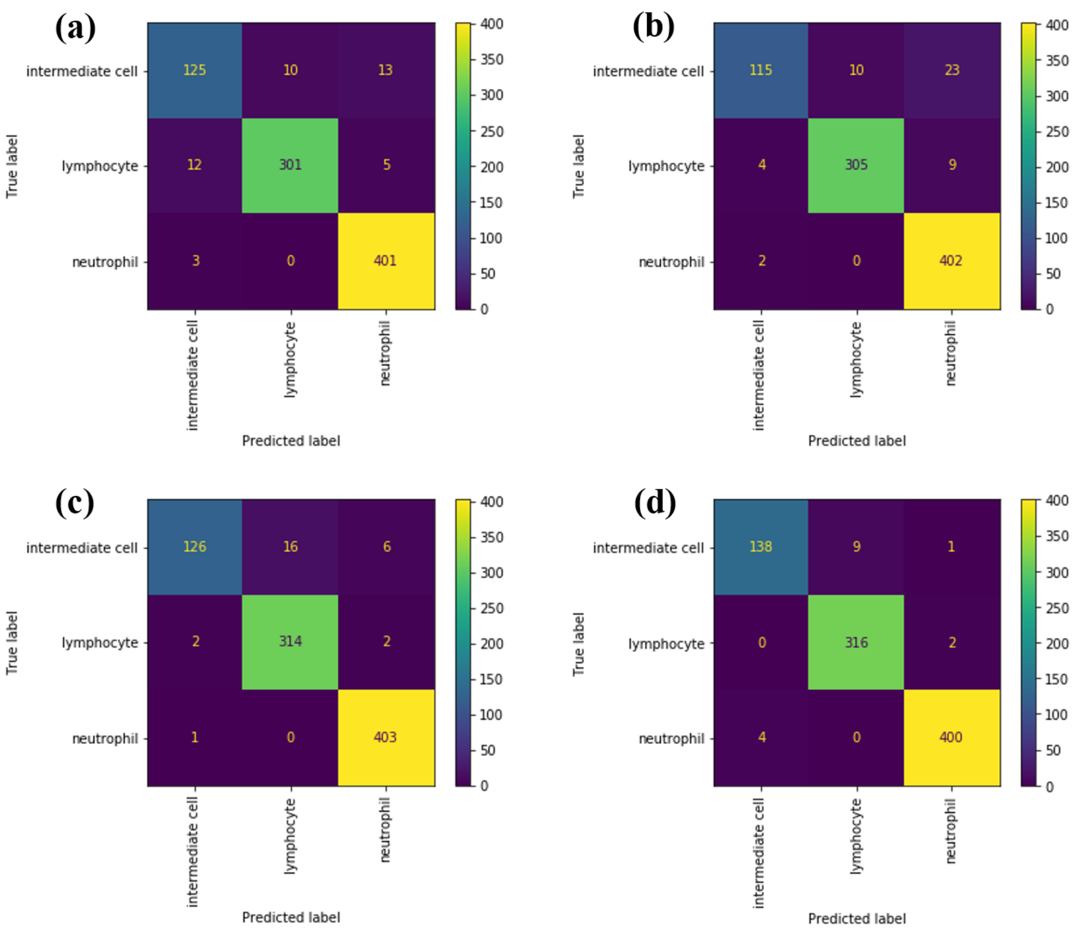

S5 Fig. Confusion matrices of WBC subtypes. (a) MobileNetV2. (b) ResNet. (c) SE-ResNeXt. (d) WBC-AMNet.

Quad-classification task

S6 Fig demonstrates the quad-classification confusion matrices for 4 CNN models in a single-cell scene. In S6 Fig the misclassification problem is almost rare. Based on the above conclusions, we can assume that WBC-AMNet demonstrates the great potential of its application with high classification accuracy.

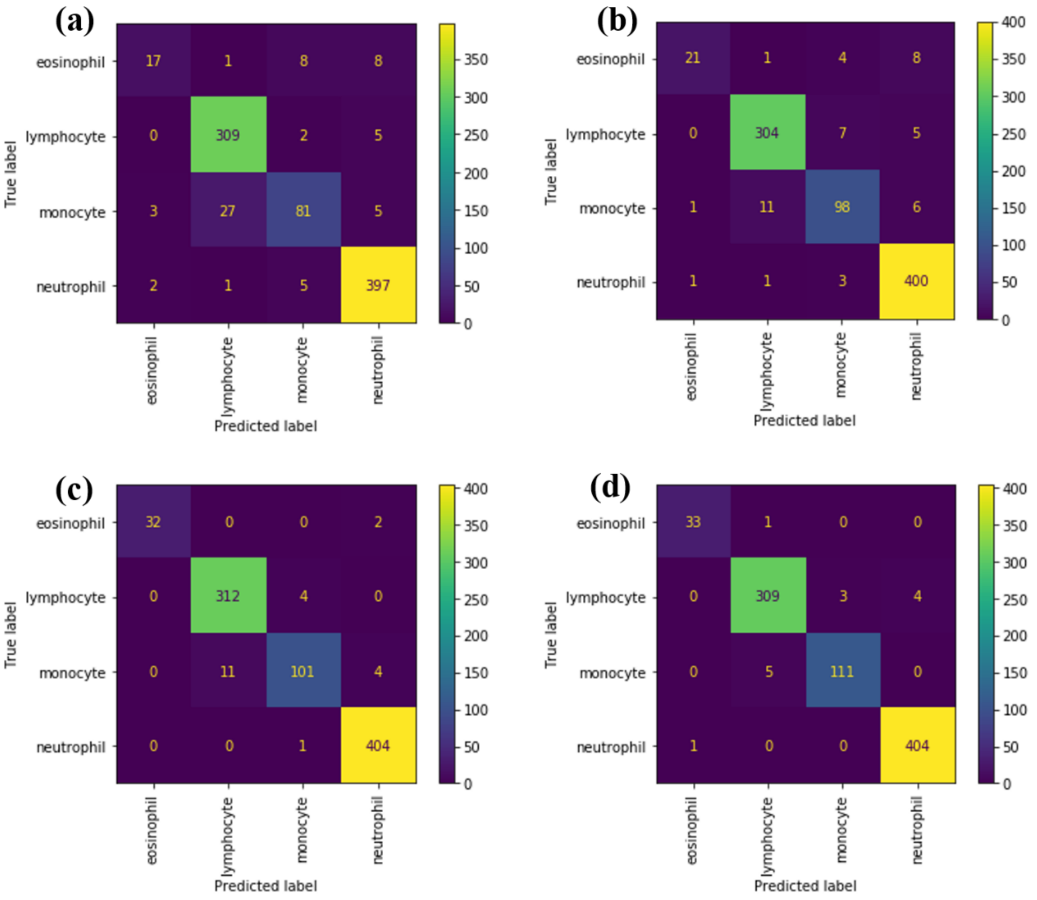

S6 Fig. Confusion matrices of WBC subtypes. (a) MobileNetV2. (b) ResNet. (c) SE-ResNeXt. (d) WBC-AMNet.
